# Applying UK real-world primary care data to predict asthma attacks in 3776 well-characterised children: a retrospective cohort study

**DOI:** 10.1038/s41533-018-0095-5

**Published:** 2018-07-23

**Authors:** Steve W Turner, Clare Murray, Mike Thomas, Annie Burden, David B Price

**Affiliations:** 1 Child Health, University of Aberdeen, Aberdeen, UK; 2 Centre for Respiratory Medicine and Allergy, Institute of Inflammation and Repair, Manchester Academic Health Science Centre, University Hospital of South Manchester, NHS Foundatin Turst, The University of Manchester, Manchester, UK; 3 Royal Manchester Children’s Hospital, Central Manchester University Hospitals NHS Foundation Trust, Manchester, UK; 4 Primary Care and Population Sciences, University of Southampton, Southampton, UK; 5 NIHR Southampton Respiratory Biomedical Research Unit, Singapore, Singapore; 6 Observational and Pragmatic Research Institute Pte Ltd, Singapore, Singapore; 7 Center of Academic Primary Care, University of Aberdeen, Aberdeen, UK

## Abstract

Current understanding of risk factors for asthma attacks in children is based on studies of small but well-characterised populations or pharmaco-epidemiology studies of large but poorly characterised populations. We describe an observational study of factors linked to future asthma attacks in large number of well-characterised children. From two UK primary care databases (Clinical Practice Research Datalink and Optimum Patient Care research Database), a cohort of children was identified with asthma aged 5–12 years and where data were available for ≥2 consecutive years. In the “baseline” year, predictors included treatment step, number of attacks, blood eosinophil count, peak flow and obesity. In the “outcome” year the number of attacks was determined and related to predictors. There were 3776 children, of whom 525 (14%) had ≥1 attack in the outcome year. The odds ratio (OR) for one attack was 3.7 (95% Confidence Interval (CI) 2.9, 4.8) for children with 1 attack in the baseline year and increased to 7.7 (95% CI 5.6, 10.7) for those with ≥2 attacks, relative to no attacks. Higher treatment step, younger age, lower respiratory tract infections, reduced peak flow and eosinophil count >400/μL were also associated with small increases in OR for an asthma attack during the outcome year. In this large population, several factors were associated with a future asthma attack, but a past history of attacks was most strongly associated with future attacks. Interventions aimed at reducing the risk for asthma attacks could use primary care records to identify children at risk for asthma attacks.

## Childhood asthma: prediction from past attacks

A past history of asthma attacks in young children is a strong predictor for future attacks and should be factored into treatment regimes. Childhood asthma attacks take considerable toll on sufferers and their carers, yet risk factors for future attacks are unclear. David Price at the Observational and Pragmatic Research Institute in Singapore and co-workers searched UK primary care databases to find at least two years’ worth of consecutive data on children with asthma aged 5 to 12. The team analyzed data from 3,776 children. Their results showed that past attacks are the strongest predictor for future attacks; of 638 patients who experienced more than one asthma attack during the first year, 240 (38%) had attacks in the second year. Other risk factors included reduced peak flow, lower respiratory tract infections and younger age.

## Introduction

There are one million children in the United Kingdom with asthma^1^ and six times as many in the United States.^2^ Asthma attacks, characterised by acute cough, wheeze and shortness of breath, are very common and affect approximately 50% of children with asthma each year.^2^ Asthma attacks result in morbidity and occasionally mortality, and also disrupt both the child’s education and their parent’s economic activity. At least one-third of healthcare expenditure on childhood asthma is spent managing asthma attacks.^3^ Understandably, preventing asthma attacks is a priority in national asthma guidelines,^4–6^ but there is surprisingly little understood about what factors are associated with future asthma attacks in children.

Good control may be a predictor of future attack,^4^ and many children are not optimally controlled,^7^ but the relationship between current control and future attacks is relatively weak^8^ in part because children are often well controlled for the majority of the time, but develop attacks with rhinovirus infection. Data from clinical trials where participants are well-characterised but relatively small in number find that risk factors for asthma attacks include a recent attack^8–10^ and clinical measurements such as blood eosinophilia,^10^ spirometry (forced expiratory volume in 1 s (FEV_1_))^11^ and bronchial hyperreactivity.^10^ Other factors associated with future asthma attacks in children include young age,^10^ ethnic group,^8^ obesity^12^ and adherence to asthma medication.^13^ Pharmaco-epidemiology studies of large unselected populations of children but who are not characterised in great detail, find that risk factors for asthma attacks, identified from prescribing or admission data, include recent treatment with oral corticosteroids,^14,15^ recent admission to hospital with asthma,^14^ increased use of bronchodilator medication^14^ and increased asthma severity.^15^ To the best of our knowledge, there are no studies which describe risk factors for future asthma attacks in the United Kingdom, where the healthcare setting is different to many countries. There are also no studies of which we are aware which relate both routinely acquired outcomes (e.g. prescribing) and clinical measurements (e.g. lung function) to risk for future asthma attacks.

Observational studies which use routinely acquired “real-world” data collected in everyday clinical practice give the opportunity to study outcomes in many conditions.^16^ Here we use a large dataset holding routinely acquired patient information to address the question “what factors available in primary care practice can be used to predict asthma attacks in children aged 5–12 years?” Predictive variables included oral corticosteroid treatment for a past attack (this was the definition of an asthma attack in the present study), current control, severity (as evidence by treatment step), age, sex, obesity, peak flow and blood eosinophilia. The latter was included in light of evidence from children^17^ and adults^18^ that eosinophilia is predictive of future asthma attacks. Although the Global INitiative for Asthma (GINA) guideline does not recommend routine measurement of eosinophil count, it does identify eosinophilia as a risk factor for asthma attacks in children aged 6–11 years and adolescents.^6^

## Results

### Study subjects

**Fig. 1:**
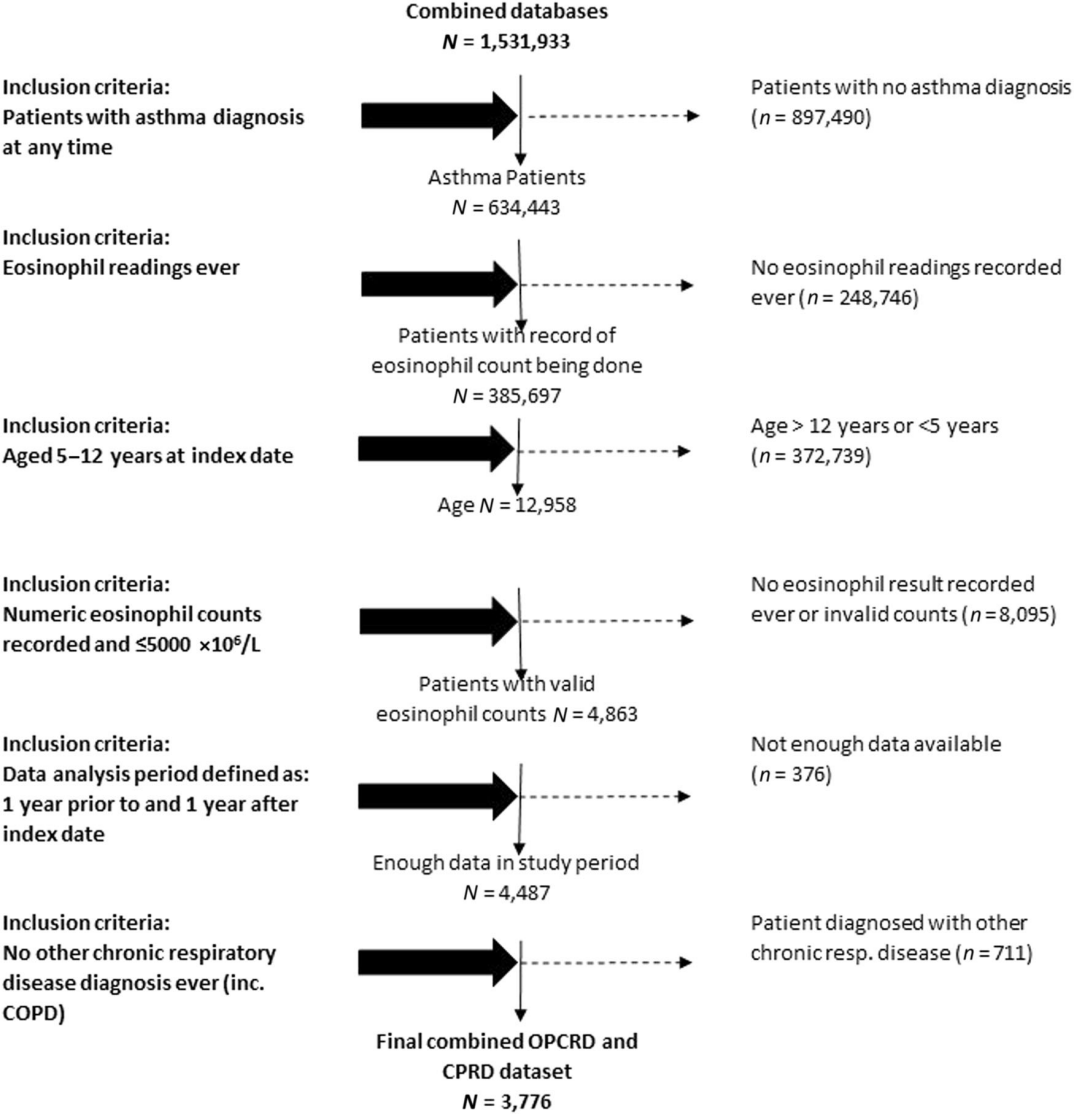
A consort-style diagram showing how children described in the present study were identified from the whole population. COPD chronic obstructive pulmonary disease

There were 3776 children identified, the mean age was 9.0 years (SD 2.3) and 57% were male. Figure 1 shows how individuals were identified from the whole population. There were 638 children (16.9%) with ≥1 attack in the baseline year, of whom 178 had ≥2 attacks in the baseline year. In the outcome year, there were 525 (13.9%) children with ≥1 attack(s) including 159 (4.2%) with ≥2 attack(s). Of the 638 patients with ≥1 attack(s) in the baseline year, 240 (38%) had at least one attack during the outcome year. For the 460 patients with exactly one attack in the baseline year, 143 (31%) had an attack during the outcome year, and for the 178 with ≥2 attacks in the baseline year, there were 97 (54%) who had one attack in the outcome year. Data were complete for all variables apart from body mass index (data available in 2037 children) and peak expiratory flow (PEF) (data available in 2216). Compared to a population of children from an Optimum Patient Care Research study who met the same inclusion and exclusion criteria except the presence of eosinophil data, the 3776 children in the present study were of similar age and were no more likely to be prescribed short-acting β-agonists (79 vs. 78%), but had a smaller proportion of boys (57 vs. 62%), were better controlled (74 vs. 61%) and were more likely to be prescribed inhaled corticosteroids (70 vs. 65%) (see Table 1).

**Table 1:** Comparison of children included in the present study with children with asthma in primary care

	Population in current study	Reference population
Mean age (SD), years	9.0 (2.3)	9.0 (2.2)
*Gender*		
% Female (*n*)	44% (1642)	38% (4213)
% Male (*n*)	57% (2134)	62% (6812)
*Overall asthma control*		
% Controlled (*n*)	74% (2768)	61% (6765)
% Uncontrolled (*n*)	26% (1008)	39% (4265)
Daily SABA dosage (μg)		
% on no SABA (*n*)	21% (777)	22% (2381)
% prescribed 1–100 (*n*)	12% (465)	51% (5572)
% prescribed 101–200 (*n*)	28% (1064)	20% (2180)
% prescribed 201–300 (*n*)	17% (647)	5% (533)
% prescribed 301+ (*n*)	22% (823)	3% (364)
Average ICS daily dose (BUD equivalent) (μg)		
% prescribed none (*n*)	30% (1134)	35% (3822)
% prescribed <100 (*n*)	24% (916)	29% (3234)
% prescribed 101–200 (*n*)	22% (812)	20% (2217)
% prescribed >200 (*n*)	24% (914)	16% (1757)

Inclusion criteria for the reference population were doctor diagnosis of asthma at any time (recorded as a Read code), 1 continuous year of practice data preceding the index date and atleast one prescription for asthma therapy within the 2 years preceding the index date (SABA, ICS, LABA, ICS/LABA combinations, LTRA). Patients were excluded if their record contained an asthma-resolved Read code or a Read code for COPD or any chronic respiratory disease other than asthma The index date for each patient was defined as the date of data extraction for their general practice. Data for each patient were assessed for 1 year preceding their index date (the study year); the full period of study ran from 27 January 2009 to 21 March 2013

### Factors detected in the baseline year associated with attack during the outcome year: univariable analysis

The following factors in the baseline year were associated with increased risk for asthma attack during the outcome year: young age, a history of hayfever diagnosis, a history of eczema diagnosis, eosinophilia, uncontrolled asthma, an asthma attack, consultation for lower respiratory tract infection, increased use of reliever medication, higher dose of inhaled corticosteroid and more severe asthma (as evidenced by Global Initiative for Asthma (GINA) treatment step) (Table 2). A PEF measurement was obtained during the baseline year for 2216 children (58.7% of the cohort) and the mean (SD) PEF reading for 1878 children with no subsequent asthma attack was 103% (26) and for the 338 with ≥1 subsequent asthma attack was 99% (28), *p* = 0.020 (Table 2). Sex and obesity were not associated with altered risk for asthma attacks (Table 2).

**Table 2:** Risk predictors from univariable analysis for at least one asthma attack defined by American Thoracic Society criteria

	Number of future attacks		Total	*p* Value
	0	≥1		
Mean age (SD), years	9.0 (2.3)	8.6 (2.4)	9.0 (2.3)	<0.001^b^
*Age group*				
5–6 years, *n* (%)	605 (18.6)	139 (26.5)	744 (19.7)	<0.001^b^
7–10 years, *n* (%)	1559 (48)	244 (46.5)	1803 (47.7)	
11–12 years, *n* (%)	1087 (33.4)	142 (27)	1229 (32.5)	
*Gender*				
Female, *n* (%)	1407 (43.3)	235 (44.8)	1642 (43.5)	0.525^b^
Male, *n* (%)	1844 (56.7)	290 (55.2)	2134 (56.5)	
*IOTF grade*				
Thin, *n* (%)	279 (16.5)	53 (15.5)	332 (16.3)	0.941^b^
Normal, *n* (%)	929 (54.8)	191 (55.7)	1120 (55)	
Overweight, *n* (%)	303 (17.9)	60 (17.5)	363 (17.8)	
Obese, *n* (%)	183 (10.8)	39 (11.4)	222 (10.9)	
*Hayfever diagnosis ever*				
Yes, *n* (%)	995 (30.6)	189 (36.0)	1184 (31.4)	0.013^b^
No, *n* (%)	2256 (69.4)	336 (64.0)	2592 (68.6)	
*Eczema diagnosis ever*				
Yes, *n* (%)	1831 (56.3)	339 (64.4)	2170 (57.5)	<0.001^b^
No, *n* (%)	1420 (43.7)	186 (35.4)	1606 (42.5)	
*Eosinophil count (x10* ^*9*^ */L)*				
≤0.4, *n* (%)	1739 (53.5)	225 (42.9)	1964 (52)	<0.001^b^
>0.4, *n* (%)	1512 (46.5)	300 (57.1)	1812 (48)	
*Overall asthma control*				
Controlled, *n* (%)	2534 (77.9)	234 (44.6)	2768 (73.3)	<0.001^b^
Uncontrolled, *n* (%)	717 (22.1)	291 (55.4)	1008 (26.7)	
*Asthma attack in baseline year*				
0, *n* (%)	2853 (87.8)	285 (54.3)	3138 (83.1)	<0.001^b^
1, *n* (%)	317 (9.8)	143 (27.2)	460 (12.2)	
≥2, *n* (%)	81 (2.5)	97 (18.5)	178 (4.7)	
*LRTI consultations resulting in script for antibiotics*				
0, *n* (%)	2910 (89.5)	421 (80.2)	3331 (88.2)	<0.001^b^
1, *n* (%)	280 (8.6)	82 (15.6)	362 (9.6)	
2+, *n* (%)	61 (1.9)	22 (4.2)	83 (2.2)	
*GINA management step*				
0, *n* (%)	616 (18.9)	52 (9.9)	668 (17.7)	<0.001^b^
1, *n* (%)	395 (12.2)	47 (9.0)	442 (11.7)	
2, *n* (%)	1726 (53.1)	258 (49.1)	1984 (52.5)	
3, *n* (%)	381 (11.7)	96 (18.3)	477 (12.6)	
4, *n* (%)	125 (3.8)	67 (12.8)	192 (5.1)	
5, *n* (%)	8 (0.2)	5 (1.0)	13 (0.3)	
*Daily SABA dosage (μg)*				
None, *n* (%)	715 (22.0)	62 (11.8)	777 (20.6)	<0.001^b^
1–100, *n* (%)	410 (12.6)	55 (10.5)	465 (12.3)	
101–200, *n* (%)	925 (28.5)	139 (26.5)	1064 (28.2)	
201–300, *n* (%)	558 (17.2)	89 (17.0)	647 (17.1)	
301+, *n* (%)	643 (19.8)	180 (34.3)	823 (21.8)	
*Average ICS daily dose (BUD equivalent) (μg)*				
None, *n* (%)	1032 (31.7)	102 (19.4)	1134 (30.0)	<0.001^b^
<100, *n* (%)	796 (24.5)	120 (22.9)	916 (24.3)	
101–200, *n* (%)	708 (21.8)	104 (19.8)	812 (21.5)	
>200, *n* (%)	715 (22)	199 (37.9)	914 (24.2)	
Percent predicted peak flow readings (%)				
*N* (% non-missing)	1878 (57.8)	338 (64.4)	2216 (58.7)	0.020^a^
Mean (SD)	102.89 (25.53)	99.06 (28.24)	102.30 (25.99)	
Median (IQR)	101.1 (86.2, 117.7)	98.3 (79.5, 117.3)	100.6 (84.8, 117.6)	
Median year at start of outcome years (IQR)	2007 (2005, 2008)	2007 (2005, 2008)	2007 (2005, 2008)	0.257^b^

*IOTF* International Obesity Task Force, *GINA* Global INitiative for Asthma, *LRTI* lower respiratory tract infection

^a^*t* test

^b^*χ*^2^ test

### Multivariable analysis

In the multivariable analysis a higher GINA management step, having any consultation for lower respiratory tract infection, an asthma attack, the presence of eosinophilia and younger age were independently associated with increased risk for asthma attack during the outcome year (Table 3). The % expected PEF was also predictive of asthma attack in the multivariable analysis of the sub-group of patients with these data available (odds ratio (OR) (95% confidence interval (CI)) 0.995 (0.990, 1.000), *p* = 0.036) (Table 4).

**Table 3:** Risk predictors for at least one asthma attack defined by the American Thoracic Society criteria

Reference category	Comparison	Odds ratio (95% CI)	*p* Value	Overall *p* value
*Blood eosinophil count*				
≤400/µL	>400/µL	1.46 (1.20, 1.78)	<0.001	<0.001
*GINA management step*				
0	1/2	1.17 (0.85, 1.62)	0.331	<0.001
	3	1.80 (1.23, 2.64)	0.003	
	4/5	2.77 (1.77, 4.33)	<0.001	
*GP consults for LRTIs*				
0	1+	1.50 (1.15, 1.96)	0.003	0.003
*Asthma attack*				
0	1	3.74 (2.92, 4.80)	<0.001	<0.001
	2+	7.72 (5.55, 10.74)	<0.001	
*Age*				
Per year of age		0.93 (0.89, 0.97)	0.001	

The results are from a multivariable analysis. The following variables were significantly associated with asthma attack in the univariate analyses but were not significant in the multivariate model: hayfever diagnosis ever, eczema diagnosis ever, overall asthma control, daily short-acting β-agonist dosage and average inhaled corticosteroid daily dose.

*GINA* Global INitiative for Asthma, *LRTI* lower respiratory tract infection

**Table 4:** Multivariable analysis of the sub-group of patients with PEF data available describing risk predictors for at least one asthma attack defined by the American Thoracic Society criteria

Reference category	Comparison	Odds ratio (95% CI)	*p* Value	Overall *p* value
*Blood eosinophil count*				
≤400/µL	>400/µL	1.48 (1.15, 1.91)	<0.001	0.002
*GINA management step*				
0	1/2	1.04 (0.67, 1.61)	0.867	<0.001
	3	1.65 (1.00, 2.73)	0.050	
	4/5	2.53 (1.43, 4.46)	0.001	
*GP consults for LRTIs*				
0	1+	1.48 (1.02, 2.14)	0.040	0.003
*Asthma attack*				
0	1	3.72 (2.73, 5.06)	<0.001	<0.001
	2+	7.81 (5.18, 11.76)	<0.001	
*% predicted PEF*				
Per 1% increase		0.995 (0.990, 1.000)	0.036	0.036
*Age*				
Per year of age		0.92 (0.87, 0.97)	0.004	0.004

The results are from a multivariable analysis. The following variables were significantly associated with asthma attack in the univariate analyses but were not significant in the multivariate model: hayfever diagnosis ever, eczema diagnosis ever, overall asthma control, daily short-acting β-agonist dosage and average inhaled corticosteroid daily dose.

*GINA* Global INitiative for Asthma, *LRTI* Lower respiratory tract infection

## Discussion

Asthma attacks are common in children and potentially preventable, and this study was designed to relate a comprehensive number of factors, which are readily available in primary care, to the risk for future asthma attacks in children aged 5–12 years. Our study considered the largest number of factors previously described and used a relatively large “real-world” population. We are not aware of another published material which describes asthma attack outcomes in a UK population. Our results indicate that, of all the outcomes collected in this large study, a past asthma attack (and especially two attacks) is likely to be the best method to identify children who might benefit from a stratified intervention aimed at reducing their risk for future asthma attacks.

Factors other than a past history of attacks which were associated with increased risk for future asthma attacks included blood eosinophilia, reduced PEF, lower respiratory tract infection and younger age, and although these associations were highly significant, they were weakly related to risk for asthma attacks and therefore not likely to be particuarly helpful in risk analysis. Current asthma control (as evidenced by short-acting β-agonist use) was related to future asthma attacks in univariable analysis but not in the multivariable analysis, and this challenges the paradigm that in children current control is a useful predictor of future attacks.^4^

The majority of the literature describing factors predictive of asthma attacks in children is based on US populations where the healthcare system is very different to the United Kingdom, but the findings of the current study are nonetheless comparable with the US literature. For example, in a secondary analysis of data in 285 6–14-year-old trial participants, Covar et al.^9^ found that those who received a course of oral corticosteroids for an asthma attack were at a twofold increased risk of an attack in the following year. A large database study of 16,250 children with asthma also found a twofold increase in risk for an attack in the year after a previous attack.^14^ In a cohort of 563 6–11 year olds with severe or difficult-to-treat asthma, Haselkorn et al.^8^ report that an attack in the previous 6 months was associated with a threefold increased risk for future attack. Finally, a study of 14,303 children with asthma living in the Netherlands also found that a past attack was the strongest predictor of future attack.^15^

In contrast with the consistent literature describing the relationship between past and future asthma attacks in children, the relationship between asthma control and future attacks is much less consistent. In our study asthma control (as evidenced by use of short-acting β-agonist) was associated with increased risk for attack in the univariable analysis, but this relationship was not seen in the multivariable analysis, perhaps due to being subsumed by treatment step. Using more than three SABA canisters was associated with a 50% increased risk for future attack in the large database study previously described^14^, but a relationship between asthma control (determined by the Children’s Asthma Control Test) and future attacks was only present in one of two other studies.^9,19^ A disconnect between asthma control and attack may be explained by different underlying factors, for example, a rhinovirus infection may commonly lead to an asthma attack,^20^ but is not likely to lead to poor control outside the setting of an attack.

In our study blood eosinophilia at the index date was associated with increased odds for an asthma attack in the following year, but the magnitude of the increase was small and is consistent with a study of 333 5–11 year olds (OR 1.52)^17^ and 12–80 year olds (OR 1.48),^18^ and this suggests a common but weak association between blood eosinophilia and future asthma attacks between ages 5 and 80 years. We do not believe that blood eosinophilia should be considered as part of any routine asthma review in children, but some subgroups (e.g. those with multiple allergies) may benefit from eosinophil monitoring.

Our study observed a link between reduced PEF and increased risk of future asthma attack, but the magnitude of the association was very small. Whilst PEF monitoring is still widely used, current guidelines do not support its routine use^4^ and there is good evidence that routine PEF monitoring does not improve asthma control.^21^ There are other objective measures such as FEV_1_,^22^ fractional exhaled nitric oxide^23^ and exhaled breath condensate^19^ which may be useful in predicting asthma attacks in children, but further evaluation of these measurements are required before they can be routinely applied.

When interpreting our data it is important to note that not all children with asthma have their blood eosinophil count measured and although we have demonstrated that the participants in this study had only relatively minor differences when compared to a population of asthmatic children in UK primary care, our results are likely to be generalisable. The children included in this analysis had a very similar burden of past asthma attacks compared to other populations.^24,25^ What we do not know is why the blood eosinophilia is being checked; if blood was taken during an asthma attack, then the relationship between blood eosinophilia and future attack may be confounded by the very presence of the initial attack, and this might strengthen any association between blood eosinophilia and future attacks, but as we have previously stated, the magnitude of this association was small and of limited clinical relevance.

A limitation to our study is that we were not able to include an index of adherence in our analysis and poor adherence is a potentially modifiable risk factor for an asthma attack. A second limitation is that we did not have an index of asthma control and we inferred control status from short-acting β-agonist prescription use. A further limitation is that data for PEF and obesity were missing for a minority of children and this may have reduced the power of the analysis, but we do not believe that these missing data have substantially affected the results since the observed associations between PEF and obesity and asthma attacks were respectively weak and non-significant.

In summary, we find that a past history of asthma attacks is the best predictor of future attacks, and that blood eosinophilia and reduced PEF do not add substantially to predicting attacks. Current guidelines^4^ recommend that a review in primary care within two working days of discharge from hospital should include checking inhaler technique, reviewing the asthma action plan and modify treatment if required, and a similar intervention could be applied after an asthma attacks not requiring hospitalisation. Research is now required to determine whether an early review after an asthma attack could reduce future asthma attacks.

## Methods

### Study design

This was a historic observational cohort study using data from the Clinical Practice Research Datalink (CPRD, https://www.cprd.com/home/) and Optimum Patient Care Research Database (OPCRD, http://optimumpatientcare.org/opcrd/). The use of data was approved by the Independent Scientific Advisory Committee of the CPRD and the Trent Multi-Centre Research Ethics Committee. A steering committee was involved in a priori definition of the study methodology (including statistical analysis plan), review of analyses and interpretation of results. Data collected during a 12-month period (“baseline year”) were linked to asthma attacks during the following years (the “outcome year”). The index date (i.e. the start of the outcome year) was the date when blood eosinophil count was determined. Variables that were linked to risk of future asthma attack were selected since they have previously been associated with asthma attacks in children; also, all but eosinophil count and peak flow have been associated with the need for more troublesome asthma (as evidenced by treatment requirement) in this population.^26^ This analysis has been registered with European Network of Centres for Pharmaco-epidemiology and Pharmacovigilance (EUPAS17985) and was approved by the Anonymised Data Ethics and Protocol Transparency Committee (approval reference ADEPT 11117).

### Data sources

CPRD provides anonymised data from 15% of all general practices in the United Kingdom, and data were available from 1 January 1999 through April 2012. OPCRD contains anonymised routine medical record data from >550 UK practices and data from 1 January 1999 to December 2012 were used for this study. The characteristics of the study population (i.e. combined CPRD and OPCRD) were compared to a population of children with asthma from OPCRD (Table 1).

### Inclusion and exclusion criteria

The inclusion criteria were as follows: aged 5–12 years (consistent with the age range for children in the British Thoracic Society/Scottish Intercollegiate Guideline Network guideline);^4^ diagnosed asthma (by Read code, see supplement); 2 years continuous records present. The only exclusion criterion was the presence of a chronic respiratory condition, for example, cystic fibrosis.

### Definitions used

The American Thoracic Society/European Respiratory Society definition of a severe asthma attack^27^ was used, that is, the occurrence of any of the following: (i) an asthma-related hospital admission, (ii) an asthma-related emergency department admission or (iii) a prescription of oral corticosteroids for 3–7 days. Overall asthma control was defined as the absence of an attack (as previously defined) in the previous year and an average daily dose of ≤200 μg salbutamol/≤500 μg terbutaline.^26^ The GINA treatment step and dose of inhaled corticosteroid at the end of the baseline period were recorded. Peak expiratory flow was standardised using equations from Rosenthal et al.^28^ Blood eosinophilia was defined as total eosinophil count >400 cells/μL. Children were categorised as obese, overweight, healthy or thin according to the International Obesity Task Force criteria^29^ using data collected closest to the end of the baseline year.

### Statistical analysis

Univariable logistic regression models were used to identify baseline measures of disease severity, patient demographics and comorbidities predictive of future attacks. The dichotomous variable indicating an attack during the outcome period (Yes/No) was used as the dependent variable, with each measure of disease severity, patient demographic and comorbidity as an explanatory variable. Those variables which showed an association (p < 0.05) with future attack were entered into a multivariable model which was step-wise reduced to produce a final list of non-collinear predictors of one or more future attacks. Two-sided statistical tests were used. Linearity was verified by categorising continuous variables, and the categorical variable was used in the final model if more appropriate than a continuous variable. Covariates were selected where the *p* value from univariate analysis was <0.05; although this stringent cut off may fail to identify variables associated with the outcome,^30^ our large sample size mitigated against this possibility. Results were presented as ORs with 95% CIs. Standard statistical software was used (SAS version 9.3 and IBM SPSS version 22).

### Data availability

The authors are not the custodians of the data and cannot make original data available but all relevant data are available from the authors. Original data are available from Clinical Practice Research Datalink (https://www.cprd.com/home/) and Optimum Patient Care (http://optimumpatientcare.org/about-us/).

## Electronic supplementary material

Supplement
