# Supplementary material for: Applying UK real-world primary care data to predict asthma attacks in 3776 well-characterised children: a retrospective cohort study
Source: NPJ Prim Care Respir Med. 2018 Jul 23;28:28. doi: 10.1038/s41533-018-0095-5 (PMC6056517; doi:10.1038/s41533-018-0095-5)
Supplement: Supplementary file 1 — Supplement [file 41533_2018_95_MOESM1_ESM.pdf]

Asthma read codes

read_code	read_term
173A.	Exercise induced asthma
66YJ.	Asthma annual review
66YK.	Asthma follow-up
66YQ.	Asthma monitoring by nurse
66YR.	Asthma monitoring by doctor
8793.	Asthma control step 0
8794.	Asthma control step 1
8795.	Asthma control step 2
8796.	Asthma control step 3
8797.	Asthma control step 4
8798.	Asthma control step 5
8B3j.	Asthma medication review
9OJA.	Asthma monitoring check done
H302.	Wheezy bronchitis
H3120	Recurrent wheezy bronchitis
H3120	Chronic asthmatic bronchitis
H33..	Asthma
H330.	Extrinsic (atopic) asthma
H3300	Extrinsic asthma - no status
H3301	Extrinsic asthma + status
H330z	Extrinsic asthma NOS
H331.	Intrinsic asthma
H3310	Intrinsic asthma - no status
H3311	Intrinsic asthma + status
H331z	Intrinsic asthma NOS
H332.	Mixed asthma
H334.	Brittle asthma
H335.	Chronic asthma with fixed airflow obstruction
H33z.	Asthma unspecified
H33z0	Status asthmaticus NOS
H33z1	Asthma attack
H33z2	Late-onset asthma
H33zz	Asthma NOS
Ua1AX	Brittle asthma
X101t	Childhood asthma
X101u	Late onset asthma
X101u	Late-onset asthma
X101x	Allergic asthma
X101y	Extrinsic asthma + attack
X101z	Allergic asthma NEC
X1020	Hay fever with asthma
X1020	Pollen asthma
X1021	Allergic non-atopic asthma
X1022	Intrinsic asthma + attack
X1024	ASA with nasal polyps

X102D	Status asthmaticus
XE0YQ	Extrinsic (atopic) asthma
XE0YR	Extrinsic asthma - no status
XE0YS	Extrinsic asthma + status
XE0YT	Intrinsic asthma
XE0YU	Intrinsic asthma + status
XE0YV	Status asthmaticus NOS
XE0YV	Severe asthma attack
XE0YW	Asthma attack
XE0YX	Asthma NOS
XE0YX	Allergic bronchitis NEC
XE0ZP	Extrinsic asthma - atopy (& ++
XE0ZR	Asthma: [intrinsic] or [late++
XE0ZT	Asthma: [NOS] or [attack]
XE2Nb	Asthma monitoring check done
XE2Nb	Asthma monitored
XM0s2	Asthma attack NOS
Xa0lZ	Wheezy bronchitis
Xa1hD	Acute exacerbation of asthma
Xa9zf	Acute asthma
XaIeq	Asthma annual review
XaIer	Asthma follow-up
XaIfK	Asthma medication review
XaIu5	Asthma monitoring by nurse
XaIu6	Asthma monitoring by doctor
XaLPE	Nocturnal asthma
Xaa7B	Chronic asthma with fixed airflow obstruction
H333.	Acute exacerbation of asthma

Eosinophil read codes

read_code	read_term
424..	Full blood count - FBC
424Z.	Full blood count NOS
42K..	Eosinophil count
42K1.	Eosinophil count normal
42K2.	Eosinopenia
42K3.	Eosinophil count raised
42KZ.	Eosinophil count NOS
42b9.	Percentage eosinophils
4E32.	Sputum: eosinophilia
D403.	Eosinophilia
D4030	Hereditary eosinophilia
D4031	Idiopathic eosinophilia
D4032	Drug induced eosinophilia
D4033	Allergic eosinophilia
D4034	Secondary eosinophilia NOS
D403z	Eosinophilia NOS
H583.	Pulmonary eosinophilia
H5831	Tropical eosinophilia
H583z	Pulmonary eosinophilia NOS
J08z5	Oral mucosa eosinoph.granuloma
X00l1	Eosinophil non-allergic rhinit
X102G	Asthmatic pulm eosinophilia
X102H	Cryptogenic pulm eosinophilia
X3009	Eosinophilic oesophagitis
X80VM	Eosinophil
Xa0kb	Tropical pulm eosinophilia
XaCJj	Percentage eosinophils

COPD read codes

read_code	read_term
H3...	Chronic obstructive pulm.dis.
H3...	Obstructive chronic bronchitis
H31..	Chronic bronchitis
H310.	Simple chronic bronchitis
H310.	Chronic catarrhal bronchitis
H3100	Chronic catarrhal bronchitis
H3101	Smokers' cough
H310z	Simple chronic bronchitis NOS
H311.	Mucopurulent chr.bronchitis
H3110	Purulent chronic bronchitis
H3111	Fetid chronic bronchitis
H311z	Mucopurulent chr.bronchit.NOS
H312.	Obstructive chronic bronchitis
H3120	Chronic asthmatic bronchitis
H3121	Emphysematous bronchitis
H3122	Acute exacerbation of COAD
H3123	Bronchiolitis obliterans
H312z	Obstructive chr.bronchitis NOS
H313.	Mixd simp+mucopur chron bronch
H31y.	Other chronic bronchitis
H31y0	Chronic tracheitis
H31y1	Chronic tracheobronchitis
H31yz	Other chronic bronchitis NOS
H31z.	Chronic bronchitis NOS
H32..	Emphysema
H320.	Chronic bullous emphysema
H3200	Segmental bullous emphysema
H3201	Zonal bullous emphysema
H3202	Giant bullous emphysema
H3203	Bullous emphysema + collapse
H320z	Chronic bullous emphysema NOS
H321.	Panlobular emphysema
H322.	Centrilobular emphysema
H32y.	Other emphysema
H32y0	Acute vesicular emphysema
H32y1	Atrophic (senile) emphysema
H32y2	MacLeod's unilateral emphysema
H32yz	Other emphysema NOS
H32z.	Emphysema NOS
H36..	Mild chron obstr pulm disease
H37..	Mod chron obstr pulm disease
H38..	Sev chron obstr pulm disease
H39..	Very severe COPD
H3y..	Chronic obstr.airway dis.OS
H3y0.	Chr obs pulm dis+ac l resp inf

H3z..	Chronic obstr.airway dis.NOS
H4640	Chronic chemical emphysema
H4641	Chemical obliter.bronchiolitis
H581.	Acute interstitial emphysema
H582.	Compensatory emphysema
Hyu30	[X]Other emphysema
Hyu31	[X]O spcf chron obs pulmon dis
X101i	Chr obs pulm dis+ac exac,unspc
X101j	Occupational chr bronchitis
X101k	Byssinosis grade 3
X101l	Bronchiolitis obliterans
X101m	Drug-induced bronchiolit oblit
X101n	Pulmonary emphysema
X101o	Pulm emphysema, alpha-1 PI def
X101p	Toxic emphysema
X101q	Congenital lobar emphysema
X101r	Scar emphysema
X102z	Bronchiolitis oblit with UIP
XE0YM	Purulent chronic bronchitis
XE0YM	Fetid chronic bronchitis
XE0YN	Bullous emphysema + collapse
XE0YO	Atrophic (senile) emphysema
XE0YP	Other emphysema NOS
XE0ZN	Chronic: [bronchitis NOS] or++
XM1Qc	Tension pneumatocoele
XaEIV	Mild chron obstr pulm disease
XaEIW	Mod chron obstr pulm disease
XaEIY	Sev chron obstr pulm disease
XaIND	End stag chron obst airway dis
XaIQg	Interstit pulmonary emphysema
XaN4a	Very severe COPD
H3y1.	Chr obs pulm dis+ac exac,unspc

Rhinitis read codes

read_code	read_term
H120.	Chronic rhinitis
H1200	Chronic simple rhinitis
H1201	Chronic catarrhal rhinitis
H1202	Chronic hypertrophic rhinitis
H1203	Chronic atrophic rhinitis
H1204	Chronic infective rhinitis
H1205	Chronic ulcerative rhinitis
H1206	Chronic membranous rhinitis
H1207	Chronic fibrinous rhinitis
H120z	Chronic rhinitis NOS
H17..	Allergic rhinitis
H170.	Allergic rhinitis - pollens
H171.	Allerg.rhinit.-other allergens
H172.	Allergic rhinitis-unsp allerg
H18..	Vasomotor rhinitis
Hyu20	[X]Oth seasonl allergc rhinits
Hyu21	[X]Other allergic rhinitis
X00kv	Rhinitis - acute
X00lA	Perennial rhinitis
XE0Xx	Chronic rhinitis
XE0Xy	Chronic catarrhal rhinitis
XE0Y5	Allergic rhinitis
XE0Y6	Allerg.rhinit.-other allergens
XE0Y7	Allergic rhinitis-unsp allerg
XE2QI	Allergic rhinitis - pollens
H17z.	Allergic rhinitis NOS

Eczema read codes

read_code	read_term
14F1.	H/O: eczema
26C4.	Nipple eczema
8HTu.	Referral to eczema clinic
A540.	Eczema herpeticum - Kaposi's
F4D30	Eczematous eyelid dermatitis
F5024	Acute eczematoid otitis extern
G831.	Varicose vein leg with eczema
G832.	Varicose vein leg+ulcer+eczema
M102.	Pustular eczema
M102.	Infectious eczematoid dermatit
M102.	Infected eczema
M111.	Atopic dermatitis/eczema
M112.	Infantile eczema
M113.	Flexural eczema
M114.	Allergic (intrinsic) eczema
M119.	Discoid eczema
M12..	Contact dermatitis/other eczem
M12z1	Eczema NOS
M12z2	Infected eczema
M12z3	Hand eczema
M12z4	Erythrodermic eczema
Myu2.	[X]Dermatitis and eczema
Myu22	[X]Exacerbation of eczema
X00iT	Eczema of external ear
X40Fx	Nipple eczema
X505L	Hand eczema
X505j	Discoid eczema
X506T	Erythrodermic eczema
XE16i	Contact eczema - eyelids
XE17A	Acute eczematoid otitis extern
XE1An	Seborrhoeic eczema
XE1Ap	Contact dermatitis/other eczem
XE1Av	Eczema NOS
Xa0p8	Eczematous eyelid dermatitis
Xa9CV	Contact eczema
XaINM	[X]Exacerbation of eczema
XaQfn	Referral to eczema clinic
XaY4o	Infected eczema
m111.	Atopic dermatitis/eczema

Rhinitis read codes

read_code	read_term
H170.	Allergic rhinitis - pollens
Hyu20	[X]Oth seasonl allergc rhinits
X00l8	Hay fever - other allergen
X00l9	Hay fever - unspec allergen
X1020	Hay fever with asthma
X1020	Pollen asthma
XE2QI	Allergic rhinitis - pollens
XE2QI	Hay fever - pollens
Xa0lX	Pollinosis

Chronic respiratory disease read codes

read_code	read_term
AD5..	Sarcoidosis
H40..	Coal workers' pneumoconiosis
H41..	Asbestosis
H410.	Pleural plaque dis.-asbestosis
H41z.	Asbestosis NOS
H42..	Silica/silicate pneumoconiosis
H420.	Talc pneumoconiosis
H421.	Simple silicosis
H422.	Complicated silicosis
H423.	Massive silicotic fibrosis
H42z.	Silica pneumoconiosis NOS
H43..	Pneumoconios.-other inorg.dust
H431.	Bauxite fibrosis of lung
H432.	Berylliosis
H433.	Graphite fibrosis of lung
H434.	Siderosis
H435.	Stannosis
H43z.	Pneumoconiosis-inorg.dust NOS
H44..	Pneumopathy-other dust inhal.
H440.	Byssinosis
H441.	Cannabinosis
H44z.	Pneumopathy-dust inhalat.NOS
H45..	Pneumoconiosis NOS
H450.	Pneumoconiosis associated/TB
H46..	Resp.disease - chemical fumes
H460.	Chemical bronchitis/pneumonit.
H460z	Chemical bronch/pneumonit NOS
H464.	Chronic chemical resp.condit.
H4641	Chemical obliter.bronchiolitis
H4642	Chemical pulmonary fibrosis
H464z	Chronic chem.resp.condit.NOS
H46z.	Chemical resp.conditions NOS
H46zz	Chemical resp.conditions NOS
H48..	Progressive massive fibrosis
H4y..	External agent lung dis.OS
H4y1.	Chronic pulm.radiation disease
H4y10	Radiation pulmonary fibrosis
H4y1z	Chronic pulm.radiation dis.NOS
H4y2.	Drg-induc interstit lung disor
H4y21	Chr drg-indc interst lung diso
H4yy.	Other ext.agent resp.condition
H4yz.	External agent resp.condit.NOS
H4z..	External agent lung dis.NOS
H57y2	Pulmonary sarcoidosis
X101U	Pleural plaque dis.-asbestosis

X103X	Progressive massive fibrosis
XE0YY	Lung disease due to ext.agents
Xa9Bw	Pneumoconioses
Xa9Bw	Occupational lung disease
H4...	Lung disease due to ext.agents
